# User requirements for an emotion-intelligent autonomous care robot in long-term care

**DOI:** 10.1007/s41999-026-01532-9

**Published:** 2026-06-22

**Authors:** Marije Harmsen, Esmee Adam, Ellen Ricke, Hanneke J. A. Smaling

**Affiliations:** 1 Department of Public Health and Primary Care, Leiden University Medical Center, Leiden, the Netherlands; 2 University Network of Care Sector Zuid-Holland, Leiden University Medical Center, Leiden, the Netherlands

**Keywords:** Robot, Emotion intelligence, User requirements, Dementia, Intellectual disability, Long-term care

## Abstract

**Aim:**

To identify user requirements and stakeholders perspectives for the development and implementation of an autonomous emotion-intelligent robot in long-term care.

**Findings:**

The to-be-developed emotion-intelligent robot must meet the user requirements regarding prerequisites, functional capabilities, and physical appearance to enhance acceptance in long-term care. While the emotion-intelligent robot was seen as promising for companionship and for providing structure to care recipients, concerns about loss of personalized ‘warm’ care and reliability underscore the need for targeted education and phased implementation.

**Message:**

Autonomous emotion-intelligent robots may add value to daily long-term care practice for geriatric clinicians by complementing human care through enhanced independence, companionship, and person-centered support, provided successful adoption is ensured through attention to user requirements, ethical considerations, and clear communication, education, and phased integration strategies.

**Abstract:**

**Purpose:**

Growing care demands in long-term care (LTC), driven by aging populations and staff shortages, challenge the delivery of person‑centered care. Autonomous emotion intelligent (EI) robots, socially assistive robots that independently perform tasks and make context-aware decisions tailored to user needs, may address these challenges by supporting daily care. This study identified key user requirements for the development and future implementation of an EI robot in LTC, exploring expectations of care recipients, family caregivers, and healthcare professionals.

**Methods:**

A qualitative design with 13 focus groups and 20 interviews was employed. Data were collected in two iterative rounds. Thematic analysis was used for the analysis.

**Results:**

Participants were 4 people with dementia (mean age: 83.0 years), 7 people with intellectual disabilities (mean age: 32.9 years), 14 family caregivers (mean age: 63.3 years), and 32 HCPs (mean age: 38.6 years). The user requirements were clustered into three themes: (1) prerequisites (e.g., robustness, ease-of-use), (2) functional requirements (e.g., provide companionship, stimulate independence), and (3) physical appearance (e.g., user-specific customization, simple interface). Participants generally viewed the EI robot positively for its potential to provide companionship and structure to care recipients, but raised concerns about loss of ‘warm’ human care and its reliability. Participants indicated that successful implementation would require clear communication, targeted education, and a structured, step-by-step rollout.

**Conclusion:**

Our findings highlight a core tension between the desire for autonomy-enhancing support and concerns about relational quality, trustworthiness, and ethical boundaries. Addressing this tension will be essential for responsible development and implementation of autonomous EI robots in LTC.

**Supplementary Information:**

The online version contains supplementary material available at https://doi.org/10.1007/s41999-026-01532-9.

## Introduction

With a growing and aging population, major challenges for long-term care (LTC) arise. The number of older, care-dependent people grows, placing a high demand for LTC in the coming decades [1]. In many countries, the demand for LTC has already exceeded the available supply [2]. Furthermore, the LTC sector faces a significant shortage of qualified personnel, driven by persistent high turnover rates [3]. The demanding nature of work, together with relatively low wages, makes it difficult to attract and retain staff [4].

Two groups with particularly high care needs are people with dementia and people with intellectual disabilities (ID), many of whom reside in LTC facilities. Worldwide, about 57 million people live with dementia, with nearly 10 million new cases each year [5]. Approximately 10% of the people with dementia live in a LTC facility although the amount of people varies per country [6, 7]. The number of people with ID is estimated to be between 80 and 234 million worldwide [8, 9]. In Europe, approximately 1.5 million people with ID live in a LTC facility [10]. Challenging behavior is highly prevalent in both groups, contributing to increased workload and absenteeism [3]. Preventing and managing such behavior is therefore of critical importance.

The increase in care demand and labor shortages affects the quality of life of care recipients and healthcare professionals (HCPs) [11]. To maintain the same quality of care in the coming decades, LTC facilities need to make changes in their care organization. One way to do this is by integrating technology into healthcare [12]. Among the emerging technologies, social assistive robots (SARs) are gaining attention for their potential to support both HCPs and care recipients.

A SAR is defined as “*a robot designed to interact with people in a natural, interpersonal manner”* [13]*.* Consequently, a SAR provides assistance via social interaction rather than physical interaction [14]. In LTC, SARs can be used to assist care recipients and HCPs with daily tasks, provide companionship and emotional support, and monitor the health status of the care recipient [15].

SARs can reduce challenging behavior and loneliness, increase social involvement, improve mood, and enhance quality of life of people with dementia [15–18]. The few small-scale studies involving people with ID show that SARs improved mood and engagement in interaction and activities [19, 20], and enhanced daily structure, increasing independence [21]. Additionally, SARs can reduce HCPs’ workload due to automation, improve quality of care recipient interaction, and reduce emotional burden [20–22].

Although current SARs show promising results, most are not yet autonomous and emotion intelligent (EI) [15], limiting their full potential. An autonomous robot is a robot that can operate and perform tasks by itself without continuous human guidance [23]. A robot is considered intelligent if it can act meaningfully and make choices based on the interaction in its environment [24]. By adapting to the care recipient’s emotional state and responding accurately, user acceptance and engagement can be enhanced, maximizing the likelihood of positive effects [15, 16, 21, 22, 25]. Emotion recognition is thus an essential feature for robots aiming to become intelligent and autonomous.

Most existing studies on SARs in dementia or ID care focus on non‑autonomous or semi‑autonomous systems, often remotely controlled, scripted, or operated by staff, and with limited or no real‑time emotion recognition capabilities. Reviews consistently note that autonomy and emotion recognition are described as future aspirations rather than empirically grounded features, and that user‑level requirements for such capabilities remain insufficiently understood [15, 18, 26]. Also, few have simultaneously examined emotion intelligence and autonomy, as well as multi-stakeholder user requirements, and implementation considerations across both dementia and ID settings. This study addresses these gaps, by employing a collaborative and inclusive development approach involving all end-users, such that the new SAR meets the needs and wishes of the end-users, which is essential for user acceptance and sustainable implementation in clinical practice [17, 26, 27].

Such integration is particularly important for clinicians, who increasingly need to understand how autonomous EI robots can support daily care. For example, EI robots may contribute to early detection of distress, support predictable daily routines, and provide interim emotional reassurance when staff is unavailable. These technologies may support person-centered care, reduce caregiver burden amid staffing shortages, and offer consistent social and emotional interaction when care resources are limited. Insights into user requirements and acceptance across diverse LTC populations help clinicians determine when such systems may be appropriate, and which robot features are necessary to ensure safe, effective, and meaningful use in everyday care.

This study aimed to 1) identify user requirements for an autonomous, EI robot for people with dementia and people with ID in LTC; 2) explore the perceptions of the end-users (i.e., people with dementia, people with ID, family caregivers, and HCPs) toward such a robot; and 3) investigate the requirements for implementation in LTC.

## Methods

### Study design

**Fig. 1:**
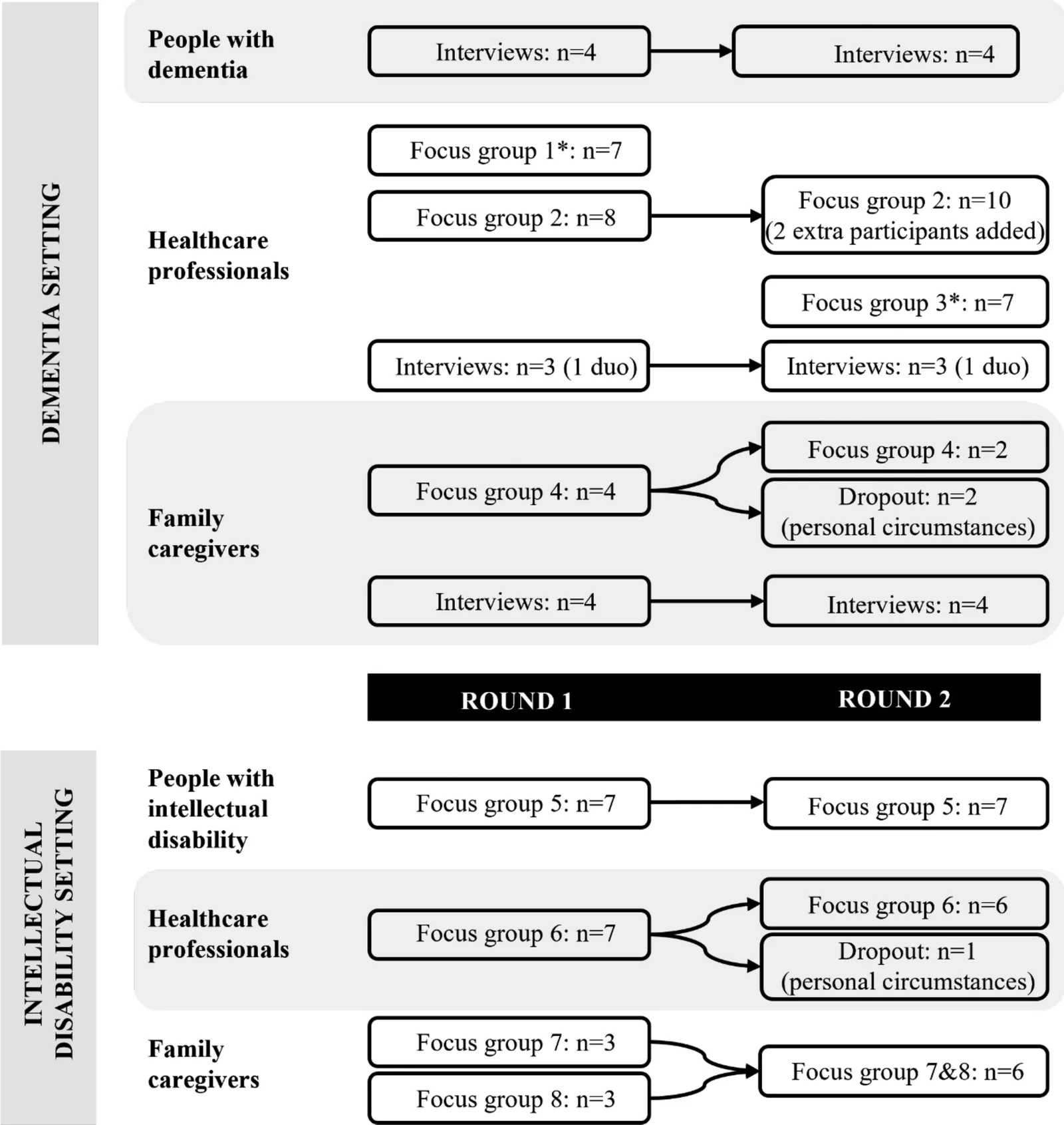
Overview of participants per data collection round per setting.* Focus group 1 and 3 were recruited via the SARA Robotics panel. Participants of those focus groups only participated in one round

A qualitative research design has been used with focus groups and interviews. Focus groups and interviews were organized per setting (dementia and ID) and per group: 1) HCPs, 2) family caregivers, 3) care recipients. The participants partook in two rounds within a six-month period to allow in-depth conversations per topic and build on the findings of previous rounds. Focus groups were chosen as the main method as they offer participants an opportunity to share their ideas, interact freely, and complement each other. Furthermore, it allowed for the content and level to be tailored to each group, introducing small nuances within the main discussion topics [28, 29]. For participants with dementia, individual interviews were conducted as they felt unsure about their ability to engage in group discussions. For other groups, interviews were offered as an alternative for those unable to attend the focus groups. Figure 1 shows an overview of the participants per round.

The separate exploration of dementia and ID settings was chosen to enhance clinical relevance, as care routines, cognitive trajectories, and ethical considerations may differ between these populations in LTC. Data were collected between June 2023 and July 2024. The Consolidated Criteria for Reporting Qualitative Research guidelines were used to report results (Supplement I) [30].

### Participants

Inclusion criteria were 1) age ≥ 18 years, 2) sufficient level of the Dutch language, and 3) ability to give consent to participate in the study. Additionally, participants with ID were included if they had mild ID (intelligent quotient (IQ) between 50 and 70) judged by HCPs or family caregivers to ensure sufficient capabilities to meaningfully participate in the focus groups. Participants with dementia were included if they were aware of their diagnosis and were considered capable of participating in an interview by their family caregiver or HCP. Family caregivers had to provide care for at least two hours per month. HCPs were included if they had ≥ 6 months of work experience with people with dementia or people with ID. Participants were excluded if their life expectancy was < 3 months.

Participants in the ID setting were recruited through a healthcare organization in the province of South Holland. Participants with dementia and their family caregivers were recruited via the University Network for the Care Sector South Holland. HCPs in the dementia setting were recruited through a LTC organization in the province of North Brabant, and the advisory panel of SARA Robotics, a company that develops care robots. Participants received an information letter, leaflet, and informed consent form. Participants could reach out to the researchers directly or give their HCP consent to share their contact details with the research team.

We used purposive sampling to recruit the stakeholder groups. Usually, 80% of all themes are found within two to three focus groups and 90% of themes are identified within three to six focus groups [31]. Moreover, in order to obtain data saturation in qualitative research, four to eight focus groups were considered as sufficient [32]. We therefore aimed for at least two focus groups per stakeholder group per setting.

### Data collection

Prior to the first round, HCPs and family caregivers received a link to an online questionnaire in Castor’s electronic data capture system [33] to ensure they met the inclusion criteria and to collect demographic data for the sample description. Care recipients filled out the questionnaire on paper before their interview or focus group and were assisted by the researchers if needed.

The focus groups took place online or at a LTC facility. Each focus group lasted between one and two hours, depending on the target group, and was moderated by HS (associate professor, female), TvH (PhD candidate, female), and EA (PhD candidate, female), who were all trained in conducting focus groups. The moderator introduced the topics and ensured that each participant could provide input and stimulate discussion. A second researcher was present to manage time and take field notes. This was done by medicine and psychology students (female: YS, male: TdK, BV, ET) who received an extensive instruction beforehand.

The interviews lasted between 30 and 60 min and were conducted by TvH and TdK. The location could be online or at a location of the interviewee’s preference. After the first interview, the interviewers received feedback from HS to ensure the quality of the interviews. The research team had no prior relation to the participants.

The focus groups and the interviews were semi-structured [34]. Minor adaptations were made to the focus group and the interview guides to tailor the level and type of questions to the target group. The guides for the focus groups and interviews were reviewed by HCPs to test the clarity and comprehensibility of the questions for people with dementia and people with ID. EI was explained as the robot’s ability to detect emotional states in a resident and initiate a tailored supportive action. Autonomy was defined as the robot’s ability to make decisions independently. For participants with dementia and participants with ID, these concepts were illustrated by concrete scenarios to support comprehension. The first round examined perceptions and functions of the EI robot, while the second round addressed its design. An overview of the discussed topics of each round together with the used materials is provided in Supplement II.

The focus groups and the interviews were audio-recorded and transcribed verbatim. A summary of the session was sent to the participants after each round to perform a member check. Additionally, researchers wrote a reflection on the session.

### Data analysis

We conducted a thematic analysis using an abductive approach, combining inductive coding informed by grounded theory principles with deductive coding based on a predefined conceptual framework [35]. An inductive approach was adopted because existing literature on user requirements and perceptions of an EI robot is scarce. This approach allowed for comprehensive, participant-driven data collection, enabling themes to emerge directly from the participants’ perspectives [35]. Subsequently, we identified inductive codes that reflected implementation factors and categorized these within relevant domains of the Consolidated Framework for Implementation Research (CFIR) [36]. The CFIR was not used to guide data collection or primary coding, but to enhance interpretive rigor and clinical relevance of the implementation findings.

Using the first three transcripts, a codebook was constructed by three researchers (HS, TvH, SW [psychology student, female]) who independently coded relevant text segments. The initial codebook was then created by comparing everyone’s codebook. Next, the consensus codebook (Supplement III) was used to re-code the same transcripts. The remaining transcripts were coded separately by two researchers (TvH, SW, BV, TdK, NK [psychology student, female], SL [male medical student], MvdB [medical student, female], NZ [medical student, female], FvZ [research assistant, female]) who all first received a training in which they coded three transcripts as practice and received feedback by HS or TvH. Frequent consensus meetings were held throughout the coding process. In case new codes merged, these were added to the codebook. HS was consulted in case of disagreement.

After all transcripts were coded, MH (junior-researcher, female) performed the analysis following the six steps of thematic analysis of Braun & Clarke [37]. During the theme development phase, HS and ER (senior-researcher, female) were consulted to provide feedback and discuss upcoming themes. All transcripts were coded using the software program ATLAS.ti version 24.2.0 [38]. Demographic data was analyzed using the software program IBM SPSS Statistics version 29.0.0 [39].

## Results

### Participant characteristics

A total of thirteen focus groups, eighteen interviews and two duo interviews were conducted. Data saturation was reached as the final interviews and focus groups did not yield any new codes. Participants included 32 HCPs, 14 family caregivers, 4 people with dementia, and 7 people with ID. Of the participants, 36 (63%) were from the dementia setting and 21 (37%) were from the ID setting. The people with dementia were living independently, of which one was admitted to a nursing home between the 1st and 2nd interviews. The people with ID were living in a LTC facility. Table 1 presents the participant characteristics.

**Table 1:** Participant characteristics

	Dementia setting			ID setting		
	People with dementia (n = 4)	Family caregivers (n = 8)	HCPs (n = 24^a^)	People with ID (n = 7)	Family caregivers (n = 6)	HCPs (n = 8)
Age (years), mean (SD, range)	83 (2.6, 80–86)	67.3 (14.4, 52–84)	40.4 (14, 18–63)	32.9 (8.1, 25–46)	60.7 (11.4, 47–78)	33.4 (10.8, 23–54)
Missing^b^		4 (50)				
Female, n (%)	2 (50)	6 (75)	23 (95.8)	4 (57.1)	4 (66.7)	6 (75)
Education level^c^, n (%)						
Low	3 (75)	2 (25)	1 (4.2)	6 (85.7)	1 (16.7)	1 (12.5)
Medium	1 (25)	–	13 (54.2)	1 (14.3)	-	4 (50)
High	–	6 (75)	10 (41.7)	-	5 (83.3)	3 (37.5)
Work experience with target group (years), mean (SD, range)	–	–	11.9 (9.4, 0.5–40)	–	–	9.1 (5.0, 3–16)
Time spent on caregiving per week (hours), mean (SD, range)	–	26.8 (57.2, 1–168)	–	–	4.1 (4.1, 1–12)	–

Type of dementia of participant or their relative, n (%)	Severity of ID of participant or their relative, n (%)	People with dementia (n = 4)	Family caregivers (n = 8)	HCPs (n = 24^a^)	People with ID (n = 7)	Family caregivers (n = 6)	HCPs (n = 8)
Alzheimer’s disease	Mild	2 (50)	3 (37.5)	–		2 (33.3)	–
Vascular dementia	Moderate	1 (25)	4 (50	–	7 (100)	2 (33.3)	–
Mixed type^d^	Profound	1 (25)	1 (12.5)	–		2 (33.3)	–
Technology experience (yes), n (%)		0 (100)	3 (37.5)	23 (95.8)	5 (71.4)	4 (66.7)	7 (87.5)
Technology experience (years), mean (SD, range)		0	14 (10.8, 5–30)	3.5 (1.8, 0.5–8)	1.8 (2.5, 0–5)	7 (5.5, 1–14)	4.1 (3.5, 1–10)
Robot experience (yes), n (%)		0	3 (37.5)	14 (58.3)	0	0	1^e^ (12.5)

*ID* intellectual disability, *HCPs* healthcare professionals, *SD* standard deviation

^a^Two healthcare professionals did not fill in the questionnaires. ^b^Four family caregivers declined sharing their age for reason of personal comfort. ^c^Low: (special) primary education, prevocational education, vocational education level 1, senior general secondary education grades 1–3, preuniversity education grades 1–3, trade school; Medium: senior general secondary education grades 4–5, preuniversity education grades 4–6, vocational education levels 2–4; High: university of applied sciences, university. ^d^Mix of Alzheimer’s disease and vascular dementia or Lewy bodies, including Parkinson’s disease. ^e^Missing data of 1 person

### User requirements for the autonomous emotion intelligent robot

Three themes were composed for the user requirements for the EI robot (see Table 2): (1) prerequisites (robustness, safety, ease-of-use), (2) functional requirements (care-related tasks, social and emotional capabilities, technical requirements), and (3) physical appearance (exterior, interface, customizability). Main themes were consistent over the settings, yet certain nuances emerged within each setting.

**Table 2:** Overview of user requirements for an autonomous emotion-intelligent robot

**Prerequisites**	
	Robust to ‘rough’ use;
	Safe, no tipping over;
	Easy to operate
**Functional Requirements**	
Care-related functions	Offer assistance in household tasks^a^;
	Offer assistance in administrative tasks^c^;
	Offer assistance to maintain independence;
	Monitor care recipients
	*Dementia Setting*
	Offer assistance in logistical tasks
Social and Emotional Capabilities	Provide companionship;
	Provide entertainment, such as cognitive activities, music, and exercise;
	Provide comfort;
	Motivate care recipients in their daily tasks and activities;
	Detect emotional distress early;
	Recognize emotions;
	Alarm healthcare professionals in unexpected and/or unsafe situations
	*Intellectual Disability Setting*
	Provide non-judgmental support;
	Act as mediator in conflict situations^a^
	*Dementia setting*
	Conversate with care recipient on same cognitive level^b^
Technical Requirements	Quick start-up of both robot and content;
	Smooth-running software updates;
	No immediate shut-down when power is lost;
	Wireless charging, to prevent pulling of the chord or tripping;
	Integration of cameras for user and emotion recognition;
	Remote control^c^;
	Limited content access for users, to prevent exposure to unsuitable material^c^
	Easy to operate volume control;
	Adjustable height;
	Autonomous navigation;
	Emergency shut-off option;
	Integration with wearables that capture physiological parameters (e.g., heart rate), enabling continuous monitoring and providing input for timely interventions (e.g., stress detection)
	*Intellectual Disability Setting*
	Ability to video-call;
	Interactive mobility functionalities, such as dancing^a^;
**Physical Appearance**	
Exterior	Soft material;
	Warm colors;
	Easy-to-clean material;
	Facial mimicry to convey emotion
	*Dementia Setting*
	Robot should have arms, so that user can hold hands
Interface	Limited amount of buttons;
	Large fonts with clear labeling;
	Buttons must have distinct colors;
	Voice control
	Different control panels for care recipient and caregivers
	*Intellectual Disability Setting*
	Buttons with pictograms
Customizability	Adjustable gender and customizable colors to match care recipient preferences;
	Adjustable interface tailored to cognitive ability of care recipient;
	Option to personalize content;
	Integration of user profile, where content and interface preferences are stored,
	Intervention adjustable to individual or group sessions^c^

^a^Only expressed by people with dementia and/or people with intellectual disabilities. ^b^Only expressed by family caregivers. ^c^Only expressed by healthcare professionals

#### Prerequisites

All participants indicated that specific essential conditions must be met when developing an autonomous EI robot to ensure functionality and usability. The EI robot must be robust to withstand accidents and ‘rough’ use, including potential collisions (while moving autonomously) and aggression toward the robot. HCPs in both settings indicated that introducing something new can elicit unexpected behavior in care recipients which may manifest in aggression directed toward the robot. For these situations, HCPs emphasized the importance of an emergency shut-down mechanism.

Furthermore, HCPs expressed the need for an easy-to-operate system, with a clear and intuitive user interface, minimal start-up times, long battery life, and adjustable height, ensuring its usability and accessibility for a diverse range of users, including care recipients with cognitive decline and users with limited technological expertise.

*“The robot should be adjustable to eye level height, as this facilitates making contact.”* [Healthcare Professional 21, Dementia Setting]

#### Functional requirements

##### Care-related functions

Participants in both settings expressed their interest in functionalities that help maintain independence, such as reminders for appointments, medication and daily tasks, and support with lightweight household chores. They strongly opposed the idea of robots performing activities of daily living, such as feeding or bathing. These tasks were considered deeply personal and best left to human caregivers. Furthermore, safety concerns were raised by HCP, for example when a care recipient would choke during dinner. Caregivers emphasized that warm person-centered care should remain the domain of HCPs.

*“I don’t want a robot touching me. I have lovely ladies who help me wash and shower. What more do I need?”* [Care Recipient 3, Dementia Setting]

*“The robot remains a machine. It doesn’t look, smell, or feel like a human. It can’t offer the warmth we provide*” [Family Caregiver 3, Dementia Setting]

HCPs highlighted the robot’s potential to reduce administrative burden through functionalities, such as voice-to-text documentation and task logging. However, a clear distinction was drawn between generating reports and interpreting them, with the latter explicitly identified as the responsibility of HCPs.

##### Social and emotional capabilities

Across both settings, participants envisioned the robot as a source of companionship and engagement for care recipients, offering cognitive and physical activities, playing music, and encouraging social and physical participation. These functions were seen as potentially freeing up time for HCPs. Emotion recognition was considered essential, enabling the robot to respond appropriately by interacting with or distracting the care recipient, or by alerting HCPs to intervene and de-escalate. The idea of the robot as a companion was emphasized particularly by family caregivers though HCPs and care recipients also acknowledged its importance.

HCPs had mixed opinions on the autonomous intervention of the robot. On the one hand, they saw potential benefits during high-stress moments, provided that responses remain simple and calming. On the other hand, they expressed caution:

*“I’m also afraid that it could trigger a shock reaction in certain clients who really need our closeness, and that such robot could actually cause tension to rise further.”* [Healthcare Professional 7, ID setting]

##### Technical requirements

Basic technical requirements should contribute to the operational reliability of the robot. Smooth operation was highlighted as critical as disruptions could cause agitation in care recipients and increase the workload.

The robot should provide autonomous navigation although it was indicated that this could also be perceived as threatening as it could be considered intrusive for certain care recipients. HCPs emphasized the need for limited access for end-users to prevent inappropriate content use which could contribute to raise tension and challenging behavior.

Opinions on using cameras and sensors for emotion and movement tracking varied. While participants recognized their value for safety and behavioral insights, concerns about privacy and data storage were raised. Data should only be retained for functional purposes and otherwise promptly deleted.

“*It’s also a piece of personal integrity. Someone is captured in their pure emotion on camera. That’s very intrusive – I understand the purpose, it can be useful, but it’s still far-reaching.*” [Family caregiver 6, ID Setting]

### Physical appearance

#### Exterior

All participants agreed that physical appearance should foster trust and comfort, avoiding an overly mechanical design. There was an inherent tension between the use of soft, tactile materials to promote comfort and the stringent hygiene requirements for LTC.

*“Color is important because of attractiveness and whether something feels threatening. Colors evoke emotions and can help establish contact.”* [Family Caregiver 6, ID Setting]

#### Interface

All participants emphasized that the robot’s interface should be simple, intuitive, and adaptable to varying cognitive and physical capabilities. This means the robot should contain a limited amount of buttons, and large fonts to ensure clear labeling. Voice control was considered beneficial, particularly for care recipients with limited motor skills or cognitive challenges. HCPs suggested combining voice control with a touchscreen and remote app, allowing staff to initiate interventions and make adjustments without direct physical contact.

HCPs stressed that certain functions, such as content restrictions or emergency shutdown, should remain hidden or require staff authorization.

#### Customizability

Customizability was viewed as essential for promoting user acceptance, enhancing engagement, ensuring interventions are effective, and providing high-quality person-centered care. For example, the interface must be adjusted to someone’s cognitive ability and preferences to prevent sensory overstimulation. Ideally, this information would be stored in a user profile. This user profile should also contain information about the end-user’s interests, history and needs.

#### Differences between dementia and intellectual disability setting

In general, there was a notable degree of similarity in the user requirements for the autonomous EI robot in both the dementia and ID setting. However, several differences emerged between both settings.

In the dementia setting, HCPs viewed the robot’s primary added value lies in supporting logistical tasks, such as inquiring about breakfast preferences or offering activities. Concerns were raised about the feasibility of emotion recognition in advanced stages of dementia, where rapid cognitive decline may increase the risk of misinterpretation and consequently increasing the distress of the care recipient.

Care recipients in the ID setting emphasized the robot’s potential to provide companionship and non-judgmental support, an aspect they perceived as difficult to achieve with human staff, who may unintentionally introduce bias. They expressed that the robot could act as a mediator in conflict situations. By comparison, people with dementia expressed mixed feelings. Some appreciated the robot’s soothing capabilities, while others doubted its ability to enable genuine two-way communication.

“*I prefer when a person comes: then you get a counter argument. You can either agree or disagree. A robot can’t do that.*” [Care Recipient 3, Dementia Setting]

Differences also emerged regarding the degree of human-likeness. HCPs in the dementia setting valued expressive features like eyes and facial mimicry to convey emotion. HCPs in the ID setting shared this view but were more accepting of a robot-like appearance. Family caregivers were cautious about overly human-like designs, fearing uncanny associations.

Several practical considerations differed across contexts. HCPs in the dementia setting highlighted that immediate shutdown could confuse care recipients and therefore recommended that the robot should announce that it is going to sleep. Furthermore, they expressed concerns that residents might impulsively press buttons during agitation and suggested temporary deactivation of controls from a distance. They also noted that tactile buttons were preferred over flat touchscreens, as people with dementia often do not intuitively engage with swipe-based interfaces.

### Perceptions of users regarding an autonomous emotion intelligent robot

The participants mentioned potential advantages and disadvantages of using an autonomous EI robot in LTC, which are presented in Table 3.

**Table 3:** Overview of perceived advantages and disadvantages of an autonomous emotion-intelligent robot

Expected advantages
Recognizes emotions in individuals who have difficulty expressing themselves^a^;
Present during times when healthcare professionals are unavailable^a^;
Facilitates communication with people who have impaired communication abilities (ID setting);
Provides immediate comfort to individuals experiencing negative emotions^a^;
Offers neutral judgment and structured interaction^a^;
Assists healthcare professional by acting as additional support, saving time and enabling personalized care^a^;
Ensures continuity in care provision despite staff changes^b^
Engages individuals who are unable to participate in group activities^c^;
Helps prevent escalation in distress-related situations^a^;
Enhances feelings of being acknowledged and provides opportunities for social interaction^a^;
Stimulates self-reliance and autonomy^b,c^ (dementia setting)
Expected disadvantages
Technical issues, such as malfunction or battery depletion^a^;
Incorrect interpretation of situations may exacerbate problems^a^;
Interventions require verification by healthcare professionals due to limited trust in technology^a^;
Increased dependency on technical developments^b^;
Requires prior knowledge of the care recipient to provide optimal interventions^a^;
Autonomous functioning necessitates camera integration and data storage^b,c^;
Risk of dehumanization of care^a^;
Environmental adjustments may be needed to accommodate the robot’s movement^c^;
Inability to intervene in unexpected situations or conversations^b,c^ (dementia setting);
Robot is not accepted by everyone.^b,c^

^a^mentioned in all participant groups; ^b^mentioned by family caregivers; ^c^mentioned by healthcare professionals

*ID* Intellectual disability

Participants indicated that the robot could have a significant impact when the EI robot recognizes emotions and can intervene accordingly.

“*The robot is an addition, not a replacement. Just an extra tool, an extra pair of eyes and ears of the healthcare professional*” [Family Caregiver 3, Dementia Setting]

Participants were positive about the social interaction the EI robot could provide, allowing the care recipients to feel seen, thereby possibly decreasing loneliness. Additionally, participants appreciated the robot providing non-judgmental responses (ID setting) and its structured communication style (both settings), noting that the robot provides predictable reactions to specific inputs.

*“You can just share your story, without judgements. The support worker will always have some sort of judgement, the robot is neutral”* [Care Recipient 3, ID Setting]

Despite these advantages, concerns were raised about the robot’s ability to properly interact with the end-user:

*“I really like it that a woman comes here to offer me some coffee. That personal connection is really important. A robot is distant. I cannot talk to the robot and tell him what is right and what is wrong. No, I don’t want to do that”* [Care Recipient 3, Dementia Setting]

HCPs worried that inappropriate interventions may escalate the situation further, and that time is needed to fully trust the judgment of the robot. A family caregiver added the concern about the increasing dependency on technology. This was confirmed in all groups, where the dehumanization of care was considered as a risk. Concerns were also expressed about the safety of integration of cameras and data storage in general.

### Facilitators for and barriers to implementation.

Family caregivers and HCPs identified multiple factors influencing the implementation of the EI robot in LTC, which were categorized using the CFIR domains (Table 4). Key barriers included limited acceptance among some care recipients and HCPs, insufficient communication across stakeholder groups, and an implementation trajectory perceived as inadequately paced. A central facilitator was the provision of targeted educational activities that contextualized the EI robot within current challenges in LTC, such as workforce shortages. This education can mitigate concerns about job displacements among HCPs and fears regarding dehumanization of care. Additionally, active involvement of stakeholders throughout the implementation process was identified as critical for successful adoption.

*“I think you need to show your loved ones what the value is. And you show that by showing (…) what my father thinks about it. That’s important to me. If my father enjoys it, then that makes me happy, and I really assume that my father will enjoy it. The alternative is that nothing happens. Because it’s not a choice between a care worker or a robot. No, it’s a robot or nothing. That’s what we’re talking about here. Now, if that robot is some kind of nasty thing that my father finds scary, then we have a different discussion. And that’s why I think it’s important to see what my father thinks about it.”* [Family Caregiver 3, Dementia Setting]

**Table 4:** Expected facilitators for and barriers to the implementation of an emotion-intelligent robot in long-term care

CFIR Category	Facilitators	Barriers
**I. Innovation domain**		
*Source (perceived credibility of the innovation developer)*	Co-create robot with end-users and stakeholders^a^	–
*Design (quality and usability of the innovation)*	EI robot meets user requirements	EI robot does not fully meet user requirements
*Relative Advantage (the innovation is better than current practice)*	Perceived enhancement of person-centered care; Perceived reduction of workload for HCPs	Robot is not suitable for all care recipients
*Adaptability (the innovation can be modified, tailored, or refined to fit local context or needs)*	Robot content can be tailored to care recipients’ cognitive levels	Limited engagement when content is mismatched to cognitive level
**II. Outer Setting domain**		
*Local Attitudes (sociocultural values and beliefs encourage the Outer Setting to support implementation)*	Family caregivers recognize added value of EI robot	–
**III. Inner Setting domain**		
*Tension for change (the current situation needs to change)*	Robot perceived as contributing to solutions for staff shortages	Resistance to change among some HCPs
*Communications (there are high-quality formal and informal information sharing practices)*	Clear communication about the robot’s purpose and added value	Insufficient communication during implementation; Lack of clarity on robot functionality and benefits
*Access to knowledge and information (guidance and training is accessible)*	Targeted education on robot operation and benefits	Insufficient explanation of working principles and added value
**IV. Individuals domain**		
*Implementation leads (Individual who lead efforts to implement the innovation)*	Dedicated innovation teams guiding implementation; Enthusiastic leaders motivating colleagues	Absence of clear ownership or responsibility for implementation
*Implementation Recipients (individual who are receiving the innovation)*	Curiosity of care recipients to interact with the robot^a^	Limited acceptance of the robot by care recipients
*Need (the individual experiences deficits which will be addressed by implementation of innovation)*	HCPs perceive added value; Reassurance that robot does not replace HCPs	Limited trust in technology by HCPs; Low acceptance of technology; Robot perceived as burdensome in practice; Job-displacement concerns unaddressed
*Motivation*	Interest among HCPs to work with the robot	Reduced motivation if feedback is not incorporated
**V. Implementation** **Process domain**		
*Engaging (attract and encourage participation in implementation)*	Early involvement of all stakeholders; Start with enthusiastic HCPs; Selecting care recipients open to technology; Involvement of family caregivers in implementation	Not involving relevant stakeholders
*Planning (identify roles and responsibilities, outline specific steps and measures for implementation success in advance)*	Sufficient time allocated for implementation	Rushed implementation; Absence of a clear, structured implementation plan
*Reflecting and Evaluating (collect and discuss measures about the success of implementation or innovation)*	Feedback actively incorporated into the implementation process	Feedback not systematically considered

*CFIR* Consolidated Framework for Implementation Research, *EI* emotion intelligent, *HCPs* healthcare professionals

^a^Reported only in dementia setting

## Discussion

This study identified three themes of user requirements for a to-be-developed autonomous EI robot in LTC: (1) prerequisites, (2) functional requirements (care-related functions, social and emotional capabilities, technical requirements), and (3) physical appearance (exterior, interface, customizability). End-users valued the robot’s potential to provide structure and companionship, while expressing persistent concerns about warmth and reliability. For successful adoption in LTC, clear communication, targeted education, and a gradual implementation process will be essential. Overall, our findings reveal a central tension between the desire for autonomy-enhancing support versus concerns about relational quality, trustworthiness, and ethical boundaries in autonomous EI robots in LTC.

End-users endorsed features that support independence (e.g., medication and task reminders) yet regarded robot‑assisted activities of daily living as inappropriate, which is in line with earlier studies [25]. This reluctance toward robot-assisted personal care may stem not only from safety concerns [40], but also from deeper normative expectations regarding the relational and moral nature of caregiving. For care recipients, robot-assisted personal care may threaten dignity and autonomy [40], whereas for caregivers it may challenge professional identity or emotional closeness resulting in worries about dehumanization of care [41]. More broadly, AI-driven technologies in LTC could further disrupt relational dynamics, underscoring the need to preserve authentic care relationships [42]. These findings support a complementary positioning of robots, while reserving hands‑on intimate care for human caregivers. Future research should examine how contextual factors influence the acceptance of robots in personal care and explore whether this acceptance evolves when robots become more integrated into healthcare.

The divided opinions on EI underscore the tension between technological promise and practical concerns in LTC. While future end-users acknowledged its potential to enhance personalized support, caregivers feared misinterpretation and its consequences. From a geriatric‑clinical perspective, EI should be positioned as a supportive capability rather than a primary decision‑maker, at least for now. We recommend human‑in‑the‑loop protocols, graded escalation to clinicians when algorithmic confidence is low, and transparent logging of detections and actions to enable learning and accountability.

Importantly, the expressed concerns cannot be approached solely as a technical challenge; it also raises normative questions about responsibility, agency, and trust in care relationships, with important implications that differ across LTC populations. In the dementia setting, concerns about emotion recognition and interaction design were closely linked to progressive cognitive decline, fluctuating emotional expression, and heightened sensitivity to confusion or overstimulation. This helps explain why participants emphasized predictability, simplified interface, and practical rather than relational robot functions. In this context, EI capabilities may be most appropriate when used conservatively, supporting daily routines and emotional reassurance while leaving complex emotional interpretation and care decisions to human caregivers. In contrast, participants in the ID setting attributed greater value to the robot’s social role, emphasizing companionship, non-judgmental support, and mediation. Here, the robot was not merely perceived as an assistive tool, but as a potential extra HCP that could provide stability and impartiality in ways that human HCPs may not always achieve. These contrasting factors underscore that EI robots cannot be designed or implemented as uniform solutions across LTC populations, but instead require alignment with cognitive, relational, and ethical characteristics of the specific care context.

Future end-users valued companionship, (cognitive/physical) activation, and enhanced self-determination provided by the robot, outcomes that relate to quality of life in LTC [15, 20, 43]. However, a clear distinction emerges between robots complementing and replacing human contact. When robots serve a complementary role, improvements in quality of life for both care recipients and caregivers were expected. Conversely, when robots substitute human interactions, concerns arise regarding authentic emotional engagement, as current robots still lack warmth and empathy, which causes resistance among all stakeholders [44]. Consequently, acceptance is expected to increase when robots assume a more complementary role in care. Future research should examine differences in attitudes and perceptions toward complementary versus substitutive roles, as well as the influence of stakeholder characteristics and contextual factors on these attitudes. To align with person‑centered care and increase adoption, messaging and deployment should explicitly frame robots as aides, not replacements. Taken together, these findings help explain why increasing technological sophistication does not necessarily lead to greater acceptance or meaningful clinical benefit in LTC.

Preferences for soft, warm materials and a non‑threatening esthetic align with evidence that robot‑initiated touch can reduce stress and increase perceived intimacy [45]. These preferences highlight the importance of embodiment in human–robot interaction, suggesting that material and sensory design features play a central role in shaping emotional reassurance and perceived safety. In LTC, however, infection‑prevention and cleaning protocols create a tangible design trade‑off. We recommend removable, washable, medical‑grade covers that preserve tactile comfort while meeting hygiene standards. The degree of human-likeness of the robot differed between the ID and dementia setting, which further shows the wide variability in people’s attitudes toward mechanical and humanoid appearance [46]. Moderate anthropomorphism (friendly face, clear eyes) may foster trust [47] while avoiding discomfort linked to excessive human‑likeness [46]. Future studies should determine the optimal level of human-like features in robots intended for LTC.

Although caregivers anticipated workload relief of an EI robot, there remains a lack of trust in technology. Previous studies have shown that trust in technology is influenced by perceived reliability, transparency and ethical considerations [48]. Simultaneously, they requested expanded control (content restrictions, remote lockouts, emergency stop), which can conflict with autonomy and risk shifting cognitive load back to staff. This autonomy paradox suggests a need for graduated autonomy—configurable autonomy levels with fail‑safes, role‑based permissions, and iterative feedback loops so that trust is earned through documented performance rather than presumed.

Customization of the EI robot emerged as key to acceptance and the likelihood of a successful implementation [18, 46]. This is especially important in cases where end-users are skeptical or reluctant to use the robot [49]. User profiles could be used for enhancing the person-centered approach of the EI robot, while limiting the burden for staff [17, 49]. However, this raises ethical questions, particularly in populations with cognitive impairment about consent, data ownership, and the dynamic alignment between recorded preferences and current wishes. Future research should focus on developing guidelines for the ethical use of EI robots in LTC with care recipients with limited decision-making capacity. Without clear guidelines, we propose dynamic consent (periodic reconfirmation with legal representatives where appropriate), data minimization, role‑specific access, and auditable deletion when retention is no longer clinically justified.

All potential facilitators and barriers identified in this study relate to a hypothetical implementation, as the autonomous EI robot is not yet available in practice. While these insights align with the CFIR framework and previous research on social robots in LTC [36, 50], they represent anticipated challenges and opportunities rather than empirical implementation outcomes. The CFIR framework remains a useful structure for conceptual planning, but the emotionally and socially interactive nature of EI robots may require extensions of the framework to adequately capture relational and ethical dimensions. Further research should explore the needs and support required during implementation for more reluctant end-users using co-design and action research methods to enhance adoption and implementation success.

From a clinical geriatric perspective, this study offers actionable guidance for the responsible integration of autonomous EI robots in LTC. Our findings indicate that clinical appropriateness depends strongly on population-specific indications and suggest distinct clinical positioning across care contexts. For people with dementia, EI robots seem best positioned when supporting structured daily routines, logistical tasks, and mild emotional reassurance, whereas for people with ID, greater clinical value may lie in companionship, predictable interaction, and non-judgmental mediation. Importantly, our findings help clinicians distinguish between supportive and substitutive robot roles, thereby safeguarding person-centered care, professional identity, and patient dignity.

### Strengths and limitations

Strengths of this study consist of the inclusion of all stakeholder groups (people with dementia, people with ID, family caregivers, and HCPs) which enriched the dataset and strengthened the robustness of the findings. Methodological rigor was ensured by combining focus groups with interviews, member checks, and systematic coding with consensus meetings.

A limitation of this study is recruitment through a single organization per setting, where most HCPs were familiar with using technology in LTC, potentially affecting perceptions, and limiting generalizability. The absence of participants with more severe dementia and participants with profound ID might have restricted the range of perspectives, a common challenge in assistive technology research [26]. Given the small number of participants with dementia, findings in these groups should be interpreted as exploratory. Future studies should involve diverse LTC settings and include individuals with varying cognitive impairments to validate and extend these findings.

## Conclusion

This study identified key user requirements and stakeholder perspectives for developing and implementing an autonomous EI robot in LTC. It is important that the EI robot meets the identified requirements so that the likelihood of user acceptance and implementation success is enhanced. For geriatric clinicians, these findings clarify when and how EI‑robots may add value in daily LTC practice, and which safeguards are necessary to prevent harm or dehumanization. Distinguishing population‑specific indications can support informed clinical decision‑making and responsible adoption. While participants valued the EI robot’s potential to enhance care recipients’ independence, provide companionship and support care processes, concerns about warmth, reliability, and ethical boundaries persist. Successful adoption will require clear communication, targeted education, and phased implementation. EI robots should be positioned as complementary tools that reinforce person‑centered care rather than replace human interaction. Addressing technical, relational, and ethical challenges—such as privacy, trust, and customization—will be critical for acceptance and sustainability. Future research should focus on co‑design approaches, graded autonomy models, and ethical frameworks to ensure responsible integration of EI robots into LTC.

## Supplementary Information

Below is the link to the electronic supplementary material.

Supplementary file1 (DOCX 49 KB)

## Data Availability

The participants of this study did not give written consent for their data to be shared publicly, sodue to the sensitive nature of the research supporting data is not available.
