# Supplementary material for: User requirements for an emotion-intelligent autonomous care robot in long-term care
Source: Eur Geriatr Med. 2026 Jun 22;17(4):2041–55. doi: 10.1007/s41999-026-01532-9 (PMC13562261; doi:10.1007/s41999-026-01532-9)
Supplement: Supplementary file 1 [file 41999_2026_1532_MOESM1_ESM.docx]

**Supplementary material**

**User requirements for an emotion-intelligent autonomous care robot in long-term care**

Marije Harmsen^1,2^, Esmee Adam^1,2^, Ellen Ricke^1,2^ & Hanneke J.A. Smaling^1,2^

Affiliations

^1^Department of Public Health and Primary Care, Leiden University Medical Center, Leiden, the Netherlands

^2^University Network of Care sector Zuid-Holland, Leiden University Medical Center, Leiden, the Netherlands

Address for correspondence

[m.harmsen@lumc.nl](mailto:m.harmsen@lumc.nl)

Journal

European Geriatric Medicine

**Supplement I. Table S1. Detailed explanation on the consolidated criteria for reporting qualitative studies (COREQ)**

**Table S1.** **Detailed explanation on the consolidated criteria for reporting qualitative studies**

|  | **Guide questions/description** | **Remarks** | **Section** |
| --- | --- | --- | --- |
| **Domain 1: research team and reflexivity** | | | |
| a) *Personal characteristics* | | | |
| 1. Interviewer/facilitator | Which author/s conducted the interview or focus group? | The focus groups were conducted by HS, EA and TvH. YS,TdK, BV and ET were have been present as an assistant to take fieldnotes and to monitor the time.  The interviews were conducted by TvH and TdK. | Methods – Data Collection |
| 2. Credentials | What were the researcher's credentials? E.g. PhD, MD | The researchers’ credentials are as follows:  PhD: ER, HS  MSc: MH, EA, TvH¸FvZ  MSc student: YS, NZ, SL, MvdB, TdK, BV, NK  BSc student: SW, ET | Methods – Data Collection, Data Analysis |
| 3. Occupation | What was their occupation at the time of the study? | PhD student: EA, TvH  Senior researcher: ER  Associate professor: HS  Junior researcher: MH  Research assistant: FvZ  MSc student medicine: YS, NZ, SL, MvdB  MSc student psychology: TdK, BV, NK  BSc student psychology: SW  BSc student health sciences: ET | Methods – Data Collection, Data Analysis |
| 4. Gender | Was the researcher male or female? | Females: MH, EA, ER, HS, TvH, YS, NK, MB, NZ, FvZ, SW  Males: TdK, BV, SL, ET | Methods – Data Collection, Data Analysis |
| 5. Experience and training | What experience or training did the researcher have? | HS, EA and TvH all followed a training focused on conducting focus groups.  TvH. YS,TdK, BV and ET received an extensive explanation on the role of the second researcher present at the focus groups. Additionally, they joined a focus group to gain insight on the procedure.  TdK joined an interview to learn the procedure. Next, he conducted an interview, which was followed by extensive feedback.  For the coding process, an initial codebook was created by HS, TvH and SW based on three transcripts. The remaining coders first received an example dataset to practice the coding process, and received feedback. | Methods – Data Collection, Data Analysis |
| b) *Relationship with participants* | | | |
| 6. Relationship established | Was a relationship established prior to study commencement? | There was no relationship with participants prior to this research. | - |
| 7. Participant knowledge of the interviewer | What did the participants know about the researcher? e*.g. personal goals, reasons for doing the research* | Participants did not know any of the researchers. However, all participants knew that the interview was for research purposes. | - |
| 8. Interviewer characteristics | What characteristics were reported about the interviewer/facilitator? e.g. *Bias, assumptions, reasons and interests in the research topic* | N/A. | - |
| **Domain 2: study design** | | | |
| a) *Theorectical framework* | | | |
| 9. Methodological orientation and theory | What methodological orientation was stated to underpin the study? *e.g. grounded theory, discourse analysis, ethnography, phenomenology, content analysis* | A thematic analysis with an abductive approach was used to analyze the data, drawing on principles of grounded theory for inductive coding while incorporating the CFIR for deductive coding. | Methods - Data Analysis |
| b) *Participant selection* | | | |
| 10. Sampling | How were participants selected? *e.g. purposive, convenience, consecutive, snowball* | Participant were selected via purposive sampling, by using pre-existing contacts in healthcare organizations. | Methods - Participants |
| 11. Method of approach | How were participants approached? e.g. face-to-face, telephone, mail, email | Participants were initially approached through an information leaflet. For those who expressed interest, more information was shared by the research team. | Methods - Participants |
| 12. Sample Size | How many participants were in the study? | In total 57 participants were recruited (in dementia setting: 4 people with dementia, 8 family caregivers and 24 healthcare professionals; in intellectual disability setting: 7 people with an intellectual disability, 6 family caregivers, and 8 healthcare professionals). | Methods – Study Design |
| 13. Non-participation | How many people refused to participate or dropped out? Reasons? | In total four people dropped out, due to personal circumstances. For one focus group three extra participants were recruited for the second round to replace two participants of the first round. | Methods – Study Design |
| c) *Setting* | | | |
| 14. Setting of data collection | Where was the data collected? e*.g. home, clinic, workplace* | Data was collected either online via Microsoft Teams, at the location of the healthcare organization, or at people with dementia’s home. | Methods – Data Collection |
| 15. Presence of non-participants | Was anyone else present besides the participants and researchers? | In focus group 3 (healthcare professionals in dementia setting) three extra people from SARA Robotics were present. They did not participate in the conversation. In every other focus group or interview there was no-one else present besides the participants and the researchers. | - |
| 16. Description of sample | What are the important characteristics of the sample? *e.g. demographic data, date* | The important characteristics of the samples were their age, gender, education background, type of dementia/severity of ID and whether participants had experience with technology and/or social robots. | Results – Participant Characteristics |
| d) *Data collection* | | | |
| 17. Interview guide | Were questions, prompts, guides provided by the authors? Was it pilot tested? | An interview guide was made and executed as planned. In the ID setting, healthcare professionals judged whether it was appropriate for people with intellectual disability, adjustments were made if needed. Details can be found in Supplement II. | Methods – Data Collection |
| 18. Repeat interviews | Were repeat interviews carried out? If yes, how many? | No repeat interviews were carried out. | - |
| 19. Audio/visual recording | Did the research use audio or visual recording to collect the data? | On-site interviews and focus groups were audio-recorded. Online interviews and focus groups were visually recorded via Microsoft Teams. | Methods – Data Collection |
| 20. Field notes | Were field notes made during and/or after the interview or focus group? | Field notes were made by the research assistant during and after every interview and focus group. | Methods – Data Collection |
| 21. Duration | What was the duration of the interviews or focus group? | The duration of the interviews ranged from 30 to 60 minutes, while the focus groups ranged from one to two hours. | Methods – Data Collection |
| 22. Data saturation | Was data saturation discussed? | Data saturation was discussed in the methodology and results section. | Methods – Participants; Results – Participant Characteristics |
| 23. Transcripts returned | Were transcripts returned to participants for comment and/or correction? | The transcripts were not returned to participants. However, detailed summaries and description of the gathered data was sent back to the participants to perform a respondent validation. | Methods – Data Collection |
| **Domain 3. Analysis and findings** | | | |
| a) *Number of data coders* | | | |
| 24. Number of data coders | How many data coders coded the data? | The initial codebook was created by three coders (HS, TvH and SW).  The remaining transcripts were coded in pairs in varying compositions by TvH, SW, BV, TdK, NK , SL, MB, FvZ, and NZ. In case there was disagreement, HS was consulted. | Methods – Data Analysis |
| 25. Description of the coding tree | Did authors provide a description of the coding tree? | For the first three transcripts, HS, TvH and SW made an individual coding tree. After this, a consensus meeting was held, and the first three transcripts were re-coded based on the consensus-coding tree. During the coding process, new codes can be added to the coding tree after they had been discussed in the consensus meetings. | Methods – Data Analysis |
| 26. Derivation of themes | Were themes identified in advance or derived from the data? | For the user requirements and perceptions, themes were derived from the data during analysis. For the implementation facilitators and barriers, themes were identified in advance following the consolidated framework for implementation research. | Methods – Data Analysis |
| 27. Software | What software, if applicable, was used to manage the data? | Castor EDC was used to manage demographic data. Atlas.ti Version 24.2.0 was used to manage the focus group and interview data. | Methods – Data Collection, Data Analysis |
| 28. Participant checking | Did participants provide feedback on the findings? | Participants received a detailed overview of the gathered data during the sessions, however, participants did not provide feedback afterwards. | - |
| b) *Reporting* | | | |
| 29. Quotations presented | Were participant quotations presented to illustrate the themes / findings? Was each quotation identified? e*.g. participant number* | Participant quotations were presented to illustrate themes and were identified by participant number and setting. | Results |
| 30. Data and findings consistent | Was there consistency between the data presented and the findings? | Yes, there was consistency between the data presented and the findings. | Results |
| 31. Clarity of major themes | Were major themes clearly presented in the findings? | Yes, major themes were clearly presented. | Results |
| 32. Clarity of minor themes | Is there a description of diverse cases or discussion of minor themes? | Yes, minor themes were discussed. | Results and Discussion |

**Supplement II. Used materials in focus groups and interviews**

Below, a detailed description of the focus group/interview procedure and materials is provided per round and topic.

**Round 1**

*Introduction of study aims and procedure*

Focus group healthcare professionals (HCPs) and family caregivers:

At the start of each focus group, the aim of the study was explained, which was to identify requirements and preferences for an autonomous emotional intelligent (EI) robot that could potentially be implemented in future long-term care (LTC) settings. Subsequently, a brief explanation of the envisioned EI-robot was provided. The EI-robot was described as a system under development that would be able to recognize emotions and stress in residents, identify situations of distress, and autonomously initiate personalized supportive action, such as playing a resident’s preferred music to calm them. It was emphasized that the robot’s actions would be adaptable to individual resident preferences. Finally, the practical aspects of the focus group were explained.

Focus group with people with intellectual disability (ID) and interview with people with dementia:

The study aim and procedure were explained using an accessible, example-based explanation. The EI-robot was described as a care robot capable of recognizing emotions and stress and responding accordingly. A concrete example scenario was presented:

*“Kim wakes up in the middle of the night and looks around anxiously. The robot detects that she appears frightened and knows that music calms her. Therefore, the robot plays calming music, after which Kim falls back asleep after a few minutes.”*

It was highlighted that robot actions would differ per person based on individual preferences. Practical aspects of the interview / focus group were then explained.

*Icebreaker to get to know each other*

Focus group HCPs and family caregivers:

Participants were invited to briefly introduce themselves by sharing their name, professional role / relation to their relative with dementia / ID, and reason for participation.

Interview with people with dementia:

Participants were asked to briefly introduce themselves.

Focus group with people with ID:

Participants were invited to introduce themselves using a playful format, during which a balloon was passed around and participants shared their name and reason for participation.

*Perceptions of an autonomous emotion intelligent (EI) robot*

Focus group HCPs and family caregivers:

The concepts of autonomy and emotional intelligence were explained. Emotional intelligence was defined as the robot’s ability to detect emotional states in residents and initiate a tailored supportive response. Autonomy was defined as the robot’s ability to make decisions independently, without direct human control. Participants were subsequently asked to discuss perceived advantages, disadvantages, and concerns related to the use of an autonomous EI-robot, as well as its potential added value in LTC.

Focus group with people with ID:

The EI-robot was similarly explained as a robot that can recognize emotions and respond accordingly. Participants were invited to discuss possible advantages, disadvantages, and concerns. Responses were written down by a supporting researcher on sticky notes and placed on a board.

Interview with people with dementia:

An EI-robot was explained as a robot capable of recognizing emotions and acting upon them. Participants were asked to share perceived advantages, disadvantages, and concerns related to such a robot.

*Functions of the EI-robot*

Focus group HCPs and family caregivers:

Participants were asked to write down potential tasks or functions for which the EI-robot could be useful, as well as tasks for which it should not be used, using sticky notes. These were placed on a board and subsequently discussed collectively. Participants were also asked about expected acceptance of the robot among different stakeholder groups, which emotions the robot should be able to recognize, and concerns regarding data use and privacy.

Focus group with people with ID and interview with people with dementia:

Participants were asked which groups should use the EI-robot, which applications might be useful, what characteristics would be required for effective functioning in a healthcare context, which emotions it should be able to recognize, which tasks it could perform, and whether acceptance by various groups was anticipated.

*Ethical considerations*

Ethical considerations were discussed only with HCPs and family caregivers.

Focus group HCPs and family caregivers:

Participants were invited to share ethical concerns that came to mind. Subsequently, a structured discussion was held using predefined statements relating to privacy, informed consent, and the human aspect of care.

**Round 2**

*Introduction and summary of findings round 1*

Focus groups with HCPs and family caregivers:

In cases where new participants were present, introductions were conducted, and practical aspects were explained. The same EI-robot example used in round 1 was repeated. A summary of the findings from round 1 was presented using a PowerPoint slides, after which participants were given the opportunity to ask questions.

Focus groups people with ID and interview with people with dementia:

Introductions and practical aspects were addressed when necessary, and the EI-robot example was repeated.

*Tailoring the EI-robot’s functions and interface to care recipients’ needs*

All focus groups:

Participants were asked how they would prefer an EI-robot intended for ID or dementia care settings to look and function. A video of approximately 2.5 minutes was shown, illustrating possible robot functionalities and interface options, including a 3D-printed care robot prototype. For participants with ID, the video was shown at half speed to enhance comprehensibility.

The 3D-printed prototype was also physically demonstrated. The prototype included two displays: an upper display showing eyes and a mouth, and a lower display functioning as a user interface of approximately 30 cm. The upper display was capable of forward and backward tilting, and four physical buttons were located beneath the displays. Participants were asked about what they liked and did not like, potential changes, preferred modes of operation, expected roles of HCPs in operating the robot, and ways to enhance user friendliness. Additionally, participants were asked who should be allowed to operate the robot and who should have access to the data.

Interview with people with dementia:

Participants were shown an A4-poster depicting six different types of care robots, which remained visible throughout the interview:

- A medium-sized, mobile care robot with a rounded, semi-humanoid design. The robot features a screen at chest height for interaction, a simplified facial display on the head, and rigid arms without articulated hands.
- A small, table-top robot with compact and playful appearance. The robot has a digital face with prominent eyes and limited movement of the arms and torso.
- A slender, mobile robot with minimalist, non-humanoid design. The robot uses a screen as its face and lacks human-like body features.
- A small humanoid robot with a clearly defined head, arms and legs. The robot is capable of standing, walking and making gestures.
- A large, mechanically oriented care robot with robust structure and articulated robotic arms.
- A highly humanoid robot with realistic body proportions and facial features, combined with visible mechanical components.

Participants were asked to describe what their ideal care robot would look like. The same 2.5-minute video shown in the focus groups was presented at half speed, followed by a demonstration of the 3D-printed prototype. Participants were asked about their opinions of the prototype, preferred operation methods, which functions should be adaptable to the user, and how user friendliness could be improved.

*Material of the EI-robot*

All Focus groups:

Participants were shown a video of approximately 4.5 minutes demonstrating possible textile materials, sensor incorporation, and examples of sensor-embedded fabrics. The video explained how sensor embedding works but did not show the different textiles. For participants with ID, the video was shown at half speed. Seven textile materials were subsequently demonstrated in person, allowing participants to touch and feel the fabrics. Participants were then asked to rank their top three preferred materials for the robot’s exterior. Various material properties (robustness, hygiene, color, softness, sustainability and the possibility to integrate one or multiple sensors) were presented, and participants were asked to rate their importance on a scale from 1 (not important) to 10 (crucial).

Interview with people with dementia:

Participants viewed the same video at half speed and were shown the textile materials. They were asked which fabric they preferred for the robot’s exterior. Material properties were discussed, and participants indicated whether each property was considered important.

*Implementation of EI-robot*

Questions regarding implementation were only discussed with HCPs and family caregivers.

Focus group HCPs and family caregivers:

Participants were asked how an EI-robot should be implemented in a LTC facility, how different stakeholders could be involved, and how enthusiasm for the robot could be fostered. They were also asked whether the robot might affect relationships between HCPs and residents, as well as relationships with family caregivers. Finally, participants were asked to reflect on boundaries of robot use and situations in which human intervention would be required.

*Ethical considerations*

In round 2, additional ethical considerations were only discussed with HCPs and family caregivers.

Focus group HCPs and family caregivers:

A structured discussion was conducted using predefined statements concerning reduced empathy among staff, potential inequality between residents, and discomfort with the use of artificial intelligence in the EI-robot.

**Supplement III. Codebook**

1. **PERCEPTIONS**
1. Benefits
   a. Extra pair of hands / eyes (when healthcare professional (HCP) is busy with other clients)
   b. Giving (extra) attention
      i. When busy / understaffed
      ii. 1-on-1 contact
   c. Providing safety and proximity (reassuring)
   d. Social contact (buddy, chatting)
   e. Preventing escalation by identifying tension in time
   f. Time savings by preventing escalation
   g. Insight into tension and (challenging) behavior (better understanding, identifying triggers) for training HCPs (and volunteers) 🡪 better care / support
   h. (Learning to) recognize stress faster (HCPs, volunteers) 🡪 better care / support
   i. Activating residents
   j. Robot has more time and patience
   k. Robot is neutral (does not judge, has no emotion)
   l. Robot is more consistent (performs actions the same way, does not get distracted)
   m. None
   n. Giving advice and support
   o. Can't get sick
2. Cons
   a) None
   b) Getting used to the robot
   c) No personal contact (not spontaneous, no recalling what happened the day before, no emotion)
   d) Lack of peer contact (colleague who helps out, sparring)
   e) Customization is time-consuming
   f) Some 'tech skill' required (more difficult for older generation or people with dementia)
   g) Aggression towards robot
   h) In case of positive stress, you might not want to intervene
   i) Not suitable for everyone
   j) Technical dependency
3. Added value
   a) None (if there is enough staff)
   b) Extra hands for HCPs (time savings, more 1-1 contact with clients)
   c) In case of staff shortages
   d) Activate/calm residents (depending on individual needs)
   e) Buddy for flex workers (helping residents get to know them)
   f) Familiar / regular face for residents (in time of personnel changes)
   g) Facilitating social contact (e.g. via app with family at a distance, sending messages)
   h) Linking with HUME for stress detection 🡪 Robot can respond
   i) Emotion recognition has added value
   j) Quality of care improved (🡪 improves quality of life of clients)
4. Concerns/challenges (other than ethical issues; skeptical, doubtful, possibly bears on the road)
   a) Fear of where technology is heading (uncertain future)
   b) Use of robot necessity due to staff shortage
   c) Robot will replace HCPs (loss of warm care)
   d) Doubt about robot capabilities (recognizing emotion; how to anticipate quickly due to physiology that builds up slowly; flexibility/applicability of the environment)
   e) Privacy (robot's assessment of privacy-sensitive information)
   f) Misinterpretation can make the situation worse
   g) Doubt about the extent of customizability to individuals preferences
   h) Distrust of what happens to data
   i) Fear that the workload will increase
   j) Conflict in opinions towards client
   k) Risks of technology (radiation, accidents)
   l) Fear of high costs
   m) Declining comprehension in people with dementia

**B. ACCEPTANCE**

1. General
   a) Unknown makes unloved
   b) Some will never accept it (non-technical/old people)
   c) Getting used to the robot takes time
   d) Acceptance if there is added value
2. Clients
   a) No acceptance expected
   b) Positive reactions to robot
   c) Mixed (partly depending on individual capabilities and needs)
   d) Evidence needed
   e) Acceptance if there is added value
   f) In co-creation with target group
   g) Gradual familiarization needed for acceptance
   h) Yes (will be accepted/ curiosity)
   i) Make acceptance measurable
   j) Greater chance if environment accepts it (HCPs, relatives)
3. HCPs
   a) Fear of being replaced by robot / threat
   b) Added value / purpose of the deployment must be clear
   c) Yes (is accepted or want to give it a chance)
   d) No acceptance expected
4. Family caregivers
   a) Possibly less acceptance if robot starts recording (video/audio)
   b) Greater chance if you involve them early
   c) No acceptance expected
   d) Yes (accepted)
   e) In case of added value/proven effect

**C. USE OF ROBOT**

1. Target group [groups, specific disorders, etc.]
   a) Quiet residents
   b) Depression
   c) Geriatric rehabilitation (doing exercises)
   d) Autism (providing structure)
   e) Severe speech/communication problems (robot more patience)
   f) Intellectual disability (ID)
   g) Dementia
   h) Aggression / agitation
   i) Assess for each resident individually
   j) Somatic
   k) People who cannot express emotions
   l) ADHD
   m) Children (daycare)
2. Functions / tasks
   a) General use
      i. Both in group settings and individually
      ii. Feedback system (input for better policy)
      iii. Preparing for moments of agitation (e.g. eating moments, shift changes)
      iv. Locate residents
      v. Robot constantly present/part of the team.
      vi. Don't know (no idea how to use it in care, satisfied with current situation)
   b) CAN take over [what can/may the robot take over from HCPs]
      i. Calming vs. stimulating
      ii. Signaling restlessness (🡪 then also offering relaxation)
      iii. Identifying unsafe situations (🡪 signaling a HCP)
      iv. Providing security / proximity
      v. Assist client with simple tasks (reminding of activities, orientation in time and place)
      vi. Giving instructions to maintain independence (showing the way to the toilet, operating the coffee machine)
      vii. Offering food and drinks
      viii. Stimulating exercise (e.g. exercise coach, running buddy, going outside)
      ix. Social interaction (small talk, speech function, keeping company)
      x. Responding to emotions
      xi. Everything
      xii. Keeping busy/entertaining
      xiii. Taking over simple care tasks from HCPs (administration, light housework)
      xiv. ADL care tasks (washing, putting on clean pants)
      xv. Observational role
      xvi. Cognitive activation/stimulation
   c) CANNOT take over [what should stay with HCPs or should the robot not do]
      i. Little that the robot is not allowed to do
      ii. Taking over tasks that the residents can still do themselves
      iii. ADL care with physical contact (washing, changing, eating)
      iv. Medical or nursing duties
      v. No response to emotion (but call in someone else)
      vi. Warm care (attention, proximity)
      vii. Facilitating eating and drinking

**D. USER REQUIREMENTS**

1. Which emotions should be recognized
2. Design (look-and-feel)
3. Functionalities (requirements, what the robot must be able to do other than tasks that it may or may not take over)
   1. Desirable what the robot should be able to do
      i. Be able to give a 'human' or warm (physical) reaction (high five, respond with facial expressions)
      ii. Using knowledge about the resident and the environment/context when deploying interventions (resident profiles, learning ability, level, facial recognition)
      iii. Responding to emotions with appropriate response (even if resident, for example, sits quietly in a corner, taking no action, only emotion recognition; including flexibility in approach and person)
      iv. Being able to locate resident(s) themselves (identifying in the room, going there themselves)
      v. Record with video and audio during activities of daily living
      vi. Robot must be able to interact
      vii. Alert + inform HCP
      viii. Informing/communicating the robot remotely (sending a message, work/daytime activity situation)
      ix. Movement (walking along)
      x. Register physiological measurements
      xi. Cuddliness
      xii. Spatial awareness (to prevent physical accidents)
      xiii. Method of charging
      xiv. Turning off the robot
      xv. Waking up residents
      xvi. Smell
   b) Desirable what the robot should not be able to do
      i. Not responding to resident's emotions
      ii. Not judging
      iii. Saving data/saving only on function
      iv. Sensor/tracker on client instead of robot constantly following resident
      v. Always recording camera images
4. Preconditions
   a) Data is well secured
   b) Robot is robust (not easily broken)
   c) Easy to clean
   d) Easy to operate/not too complex
   e) Safe to use (e.g. suction cup against tipping over)
   f) Does not make too much noise
5. User-friendliness
a) Accessible for large group vs individual
b) Testing/adapting per target group (for optimal user-friendliness)
c) Software related (updates + fast boot)
d) Operating Instructions

**E. ETHICAL CONSIDERATIONS**

*As ethical considerations were outside the scope of this study, detailed codes have been removed from this codebook.*

1. None
2. Privacy
3. Consent if client lacks capacity
4. Human aspect
5. Care gap (choice between no care vs. care by robot; helping out family)
6. Autonomous robot
7. Risks / safety
8. Laws and regulations

**F. IMPLEMENTATION** (facilitating and hindering factors, tips)

1. Important to get family involved
2. Involving stakeholders at an early stage
3. Good information and education (including added value, workshop; including feedback results to HCPs)
4. Good guidance needed during implementation (guiding clients through care when getting acquainted)
5. Motivate HCPs and/or loved ones about use
6. National campaign technology as part of regular care
7. Granting/taking time for implementation (takes time, difficulty with changes)
8. Don't focus on those who will never accept (certain people)
9. Culture change necessary 🡪 to do it together, including robot
10. Start with capable target group to detect errors.
11. Identifying resistance and processing feedback
12. Just put it down

**G. CONTEXT**

1. Background information participant
   a) Experience, work setting
   b) Attitude towards digitalization
2. Experience with care robot
3. Experience with HUME
